# Effects of SMYD2‐mediated EML4‐ALK methylation on the signaling pathway and growth in non‐small‐cell lung cancer cells

**DOI:** 10.1111/cas.13245

**Published:** 2017-06-22

**Authors:** Rui Wang, Xiaolan Deng, Yuichiro Yoshioka, Theodore Vougiouklakis, Jae‐Hyun Park, Takehiro Suzuki, Naoshi Dohmae, Koji Ueda, Ryuji Hamamoto, Yusuke Nakamura

**Affiliations:** ^1^ Section of Hematology/OncologyDepartment of MedicineThe University of ChicagoChicagoIllinoisUSA; ^2^ State Key Laboratory of Cancer Biology and Xijing Hospital of Digestive DiseasesXijing HospitalFourth Military Medical UniversityXi'anChina; ^3^ Biomolecular Characterization UnitRIKEN Center for Sustainable Resource ScienceWakoJapan; ^4^ Cancer Proteomics GroupCancer Precision Medicine CenterJapanese Foundation for Cancer ResearchTokyoJapan; ^5^ Division of Molecular Modification and Cancer BiologyNational Cancer Center Research InstituteTokyoJapan

**Keywords:** EML4‐ALK, kinase inhibitor, methylation, NSCLC, SMYD2

## Abstract

A specific subtype of non‐small‐cell lung cancer (NSCLC) characterized with an *EML4‐ALK* fusion gene, which drives constitutive oncogenic activation of anaplastic lymphoma kinase (ALK), shows a good clinical response to ALK inhibitors. We have reported multiple examples implying the biological significance of methylation on non‐histone proteins including oncogenic kinases in human carcinogenesis. Through the process to search substrates for various methyltransferases using an *in vitro* methyltransferase assay, we found that a lysine methyltransferase, SET and MYND domain‐containing 2 (SMYD2), could methylate lysine residues 1451, 1455, and 1610 in ALK protein. Knockdown of SMYD2 as well as treatment with a SMYD2 inhibitor in two NSCLC cell lines with an *EML4‐ALK* gene significantly attenuated the phosphorylation levels of the EML4‐ALK protein. Substitutions of each of these three lysine residues to an alanine partially or almost completely diminished *in vitro* methylation of ALK. In addition, we found that exogenous introduction of EML4‐ALK protein with the substitution of lysine 1610 to an alanine in these two cell lines reduced the phosphorylation levels of AKT, one of the downstream oncogenic molecules in the EML4‐ALK pathway, and suppressed the growth of the two cell lines. We further showed that the combination of a SMYD2 inhibitor and an ALK inhibitor additively suppressed the growth of these two NSCLC cells, compared with single‐agent treatment. Our results shed light on a novel mechanism that modulates the kinase activity of the ALK fused gene product and imply that SMYD2‐mediated ALK methylation might be a promising target for development of a novel class of treatment for tumors with the ALK fused gene.

Post‐translational modifications play critical roles in protein functions by affecting protein folding, oligomerization, subcellular localization, and protein stability.^1^, ^2^ Lysine methylation on histone proteins is considered one of the most fundamental modifications for orchestrating global gene transcription.^3^, ^4^, ^5^, ^6^, ^7^, ^8^ In addition, methylation on non‐histone proteins has been shown to be dynamically regulated by protein lysine methyltransferases, protein arginine methyltransferases (PRMTs), and demethylases in the last decade. We and others have reported that aberrant methylation of oncoproteins and tumor suppressor proteins plays important roles in development/progression processes of a variety of human cancers.^9^, ^10^ Our previous studies indicated that methylation on non‐histone proteins affected subcellular localization, stability, and other modifications of several oncogenic proteins;^9^, ^10^, ^11^, ^12^ for example, methylation of heat shock protein 70 by SETD1A promotes its nuclear translocation and interaction with Aurora kinase B.^11^ We have also shown that demethylation of lysine residues in MYPT1, a retinoblastoma 1 phosphorylation regulator, by lysine‐specific histone demethylase 1 destabilizes the MYPT1 protein and promotes cell cycle progression in cancer cells.^12^ Other groups showed that methylation of lysine 372 of p53 by SETD7 increases protein stability and enhances p53 activity,^13^ whereas monomethylation of lysine 370 by SMYD2 represses p53 function.^14^ Furthermore, the activities of several protein kinases were shown to be under the control of lysine methylation; methylation of vascular endothelial growth factor receptor 1^15^ and AKT1^16^ by SMYD3 leads to enhancement of autophosphorylation. SMYD2 negatively regulates activity of the tumor suppressor phosphatase and tensin homolog and leads to aberrant activation of the AKT pathway.^17^

Activation of ALK protein, which is caused by chromosomal translocations,^18^, ^19^, ^20^ gene amplification,^21^, ^22^, ^23^ or point mutations,^22^, ^24^ plays a critical role in a small subset of NSCLC. An *EML4*‐*ALK* fusion gene generated by inversion of the short arm of chromosome 2 is observed in approximately 5% of human NSCLCs.^18^, ^25^ The EML4‐ALK fusion oncoprotein requires an N‐terminal coiled‐coil domain of EML4 which is essential for dimerization of the fusion protein and constitutive activation of ALK kinase.^26^ Tyrosine kinase inhibitors binding to an ATP‐binding pocket of ALK, such as crizotinib,^27^ ceritinib,^28^, ^29^, ^30^, ^31^ and alectinib,^32^, ^33^ have been proven their clinical effectiveness for NSCLC with genetic alterations causing aberrant ALK activation. It was also reported that SUMOylation and glycosylation on the NPM‐ALK rearranged oncoprotein affected the stability and phosphorylation of the fused protein in neuroblastoma.^34^, ^35^ However, there has been no report indicating post‐translational EML4‐ALK methylation that may affect the oncogenic activity of this fusion protein.

In the present study, through screening with the *in vitro* methyltransferase assay and LC‐MS/MS analysis, we identified that lysine residues 1451, 1455, and 1610 in an ALK tyrosine kinase domain were likely to be methylated by SMYD2. We further showed that exogenous introduction of EML4‐ALK protein with K1610A substitution into two NSCLC cell lines with endogenous EML4‐ALK protein dominant‐negatively suppressed the growth of these two cell lines. Our results imply the significant role of SMYD2‐mediated EML4‐ALK methylation in lung carcinogenesis.

## Materials and Methods

### Cell lines

Human NSCLC cell lines, H3122 and H2228, which have variant 1 and variant 3 of an *EML4‐ALK* fused gene, respectively, and the human embryonic kidney fibroblast cell line 293T were purchased from ATCC (Manassas, VA, USA) and were tested for authentication by DNA profiling with polymorphic short tandem repeat markers (Table S1). Four NSCLC cell lines without the *EML4‐ALK* fused gene were purchased from ATCC (for NCI‐H1373, NCI‐H23, and NCI‐H522) or Japanese Collection of Research Bioresources Cell Bank (Suita, Japan) (for VMRC‐LCD). 293T cells were cultured in DMEM and the six NSCLC cell lines were grown in monolayers in RPMI‐1640 medium supplemented with 10% FBS and 1% antibiotic/antimycotic solution (Sigma‐Aldrich, St. Louis, MO, USA). The cells were maintained at 37°C in humid air with 5% CO_2_.

### Mass spectrometry analysis

The ALK samples reacted with BSA or SMYD2 *in vitro* were separated by SDS‐PAGE and stained with Simply Blue Safe Stain (Thermo Fisher Scientific, Waltham, MA, USA). The ALK bands were excised and digested in gel with trypsin L‐(tosylamide‐2‐phenyl) ethyl chloromethyl ketone (TPCK‐treated; Worthington Biochem, Lakewood, NJ, USA) or endoproteinase Asp‐N (Roche Applied Science, Branford, CT, USA). Then the digest peptides were analyzed by nano LC‐MS/MS using a Q Exactive mass spectrometer (Thermo Fisher Scientific, Waltham, MA, USA). The peptides were separated using nano ESI spray column (75 μm [ID] × 100 mm [L], NTCC analytical column C18, 3 μm; Nikkyo Technos, Tokyo, Japan) with a linear gradient of 0–35% buffer B (100% acetonitrile and 0.1% formic acid) at a flow rate of 300 nL/min over 10 min (Easy nLC; Thermo Fisher Scientific). The mass spectrometer was operated in the positive ion mode, and the MS and MS/MS spectra were acquired in a data‐dependent TOP10 method. The MS/MS spectra were searched against the in‐house database using local MASCOT server (version 2.5; Matrix Sciences, Boston, MA, USA). For the quantitative analysis *in vivo* methylation, ALK peptides were monitored using targeted MS/MS method.

### Plasmid construction of substituted proteins

The pcDNA3‐*EML4‐ALK* variant 1 with N‐FLAG was kindly provided from Professor Hiroyuki Mano at The University of Tokyo (Tokyo, Japan). Using the pcDNA‐N‐FLAG‐tagged EML4‐ALK, we constructed lysine‐to‐alanine substituted plasmid clones using primers purchased from Sigma‐Aldrich (sequence is shown in Table S2) and KOD Xtreme Hot Start DNA Polymerase (Novagen, Madison, WI, USA) according to manufacturer's protocols.

### *In vitro* methyltransferase assay

A C‐terminal portion of ALK (a.a. 1058–1620) including a TKD was subcloned and ligated with pCAGGSn‐C‐3FLAG. The 293T cells were transfected with WT ALK‐TKD expression vector (pCAGGSn‐3FLAG–ALK‐TKD) or substituted types of ALK‐TKD expression vector (pCAGGSn‐3FLAG–ALK‐TKD K1451A, K1455A, or K1610A). After 48 h of the culture, the protein was immunoprecipitated with anti‐FLAG antibody and purified with Amicon Ultra Centrifugal Filters (Millipore, Billerica, MA, USA). For the *in vitro* methyltransferase assay, recombinant ALK‐TKD‐WT, ALK‐TKD‐K1451A, ALK‐TKD‐K1455A, or ALK‐TKD‐K1610A was separately incubated with SMYD2 enzyme using 2 μCi S‐adenosyl‐l‐[methyl‐^3^H]‐methionine (PerkinElmer, Waltham, MA, USA) as the methyl donor in a mixture of 10 μL methylase activity buffer (50 mM Tris‐HCl at pH 8.8, 10 mM DTT, and 10 mM MgCl_2_) for 2 h at 30°C. Proteins were resolved on a Mini PROTEAN TGX Precast gel (Any kD; Bio‐Rad, Hercules, CA, USA) and visualized by fluorography using EN3HANCE Spray Surface Autoradiography Enhancer (PerkinElmer). Loading proteins were visualized by MemCode Reversible Protein Stain (Thermo Fisher Scientific).

### Antibodies

The following primary antibodies were used; anti‐FLAG antibody (mouse, M2; Sigma‐Aldrich; dilution used in WB, 1:1000), anti‐HA antibody (rat, #11867423001; Roche Applied Science; dilution used in immunocytochemistry (ICC): 1:1000), anti‐SMYD2 antibody (rabbit, D14H7; Cell Signaling Technology; dilution used in WB, 1:1000), anti‐ALK antibody (rabbit, D5F3; Cell Signaling Technology, Danvers, MA, USA; dilution used in WB, 1:1000), anti‐phospho‐Y1604‐ALK antibody (rabbit; Cell Signaling Technology; dilution used in WB: 1:1000), anti‐phospho‐Y1278/1282/1283‐ALK antibody (rabbit; Cell Signaling Technology; dilution used in WB, 1:1000), anti‐phospho‐S473‐AKT antibody (rabbit; Cell Signaling Technology; dilution used in WB, 1:500), and anti‐β‐actin antibody (mouse, 8H10D10; Cell Signaling Technology; dilution used in WB, 1:1000).

### Western blot analysis

Samples were prepared from the cells lysed with CelLytic M mammalian cell lysis/extraction reagent (Sigma‐Aldrich) containing a complete protease inhibitor cocktail (Roche Applied Science) and a phosphatase inhibitor cocktail (Roche Applied Science), and whole cell lysates or immunoprecipitation (IP) products were transferred to 0.45 μm PVDF membrane after electrophoresis. Protein bands were detected by incubating with HRP‐conjugated antibodies (GE Healthcare, Chicago, IL, USA) and visualizing with Enhanced Chemiluminescence (GE Healthcare).

### Immunoprecipitation

SMYD2 was knocked down in H3122 cells using specific siRNAs. After 96 h of incubation, the cells were lysed with CelLytic M mammalian cell lysis/extraction reagent containing a complete protease inhibitor and a phosphatase cocktail. Whole cell extract was incubated with anti‐ALK antibody overnight. The next day, we added 30 μL/well of Protein A Sepharose beads (Invitrogen, Carlsbad, CA, USA) to the cell extract and incubated at 4°C for 1 h. After the beads were washed three times in 1 mL PBS buffer, we added 30 μL/well of Lane Marker Reducing Sample Buffer and denatured the samples, followed by Western blot analysis.

### Small interfering RNA transfection

Small interfering RNA oligonucleotide duplexes were purchased from Sigma‐Aldrich for targeting the human SMYD2 transcript. siRNA negative control (siNC) was used as a control siRNA. The siRNA sequences are described in Table S3. Small interfering RNA duplexes (final concentration, 100 nM) were transfected into H3122 and H2228 cells with Lipofectamine RNAiMax Reagent (Thermo Fisher Scientific).

### *In vitro* growth inhibition

LLY‐507, a SMYD2‐specific inhibitor, was purchased from Sigma‐Aldrich. Crizotinib was purchased from (Cayman, Ann Arbor, MI, USA). H2228 and H3122 cells were treated with either or both LLY‐507 and crizotinib at the IC_50_ concentration; we calculated that the IC_50_ values of LLY‐507 for H2228 and H3122 cells were 2 and 3 μM, respectively, and the IC_50_ values of crizotinib for H2228 and H3122 cells were 0.6 and 0.15 μM, respectively. Protein was harvested after 6 h to examine an early change of the EML4‐ALK pathway. After incubation with the inhibitor(s) for 48 h, cells were treated with CCK8, Dojindo (Kumamoto, Japan) (10 μL/100 μL) medium for 3 h in the incubator, and then measured the absorbance at 450 nm using a microplate reader. Growth inhibition rates by the inhibitor(s) on cancer cells were calculated by comparison with control cells. Experiments were done in triplicate.

### Statistical analysis

Statistical analyses were carried out using Student's *t*‐test and results are shown as the mean ± SD of three independent experiments. We considered statistically significant when *P*‐values were <0.05.

## Results

### SMYD2 methylates lysine residues in the C‐terminal TKD of ALK

**Figure 1:**
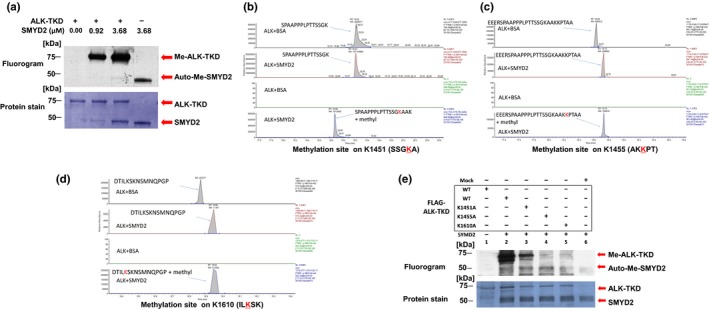
SMYD2 methylates anaplastic lymphoma kinase (ALK) *in vitro*. (a) Recombinant ALK–tyrosine kinase domain (TKD) protein was incubated with different concentrations of SMYD2 in the presence of S‐adenosyl‐l‐[methyl‐3H]‐methionine. Methylation signals were detected by autoradiography in a dose‐dependent manner (upper panel). Amounts of loading proteins were evaluated by staining with Mem Code Reversible Protein Stain (Thermo Fisher Scientific; lower panel). On an autoradiography panel, the upper bands at molecular weight ~75 kDa correspond to methylation signals of ALK‐TKD, and the lower bands at molecular weight ~50 kDa are automethylation signals of SMYD2. Lower panel, the upper and lower bands correspond to ALK‐TKD and SMYD2 proteins, respectively. (b–d) Selected full mass spectrometry ion chromatograms of unmodified and mono‐methylated ALK‐TKD peptides corresponding to methylation on K1451, K1455, and K1610, respectively, in the liquid chromatography–tandem mass spectrometry. (e) WT ALK‐TKD or K1451A‐, K1455A‐, or K1610A‐substituted ALK‐TKD proteins was incubated with SMYD2 in the presence of S‐adenosyl‐l‐[methyl‐3H]‐methionine. Methylation signals were detected by autoradiography (upper panel). Amounts of loading proteins were evaluated by staining with Mem Code Reversible Protein Stain (lower panel). On an autoradiography panel, the upper bands at molecular weight ~75 kDa correspond to methylation signals of ALK‐TKD, and the lower bands at molecular weight ~50 kDa are automethylation signals of SMYD2. Lower panel, the upper and lower bands correspond to ALK‐TKD and SMYD2 proteins, respectively.

To explore whether ALK serves as a potential substrate of any protein lysine methyltransferases or PRMTs, we undertook the *in vitro* methyltransferase assay with our recombinant methyltransferase panel (SMYD2, SMYD3, PRMT1, PRMT5, SUV39H2, WHSC1, WHSC1L1, and EZH2) and a recombinant protein corresponding to a TKD of ALK using S‐adenosyl‐l‐[methyl‐^3^H]‐methionine as a methyl resource. As shown in Figure 1(a), SMYD2 methylated ALK‐TKD in a dose‐dependent manner, confirming the *in vitro* methylation of ALK by SMYD2. Then, to identify a candidate methylated site(s), we subjected recombinant ALK‐TKD for LC‐MS/MS after *in vitro* methylation of ALK‐TKD with SMYD2 and found an increase in the molecular weight of lysine residues at codons 1451, 1455, and 1610 (these numbers are based on the positions in WT full‐length ALK protein) (Fig. 1b–d).

To further confirm SMYD2‐mediated methylation at these three residues, we generated four plasmid clones designed to express FLAG‐tagged ALK‐TKD proteins, WT ALK, and a lysine‐to‐alanine substituted ALK‐TKD at K1451 (K1451A), K1455 (K1455A), or K1610 (K1610A). After immunoprecipitation of these FLAG‐tagged proteins, we carried out *in vitro* methyltransferase assay and found that all three ALK‐TKD proteins with the substitution of a lysine residue showed partial or strong impairment of methylation of the ALK‐TKD protein (Fig. 1e). Although we were unable to conclude that the decrease of methylation levels is caused by the direct effect on the methylation site(s) or the indirect effect by the structural changes of the protein by substitution of the lysine residue, the substitution of K1455 and K1610 seemed to affect more significant ALK‐TKD methylation by SMYD2.

### SMYD2 regulates phosphorylation of EML4‐ALK

**Figure 2:**
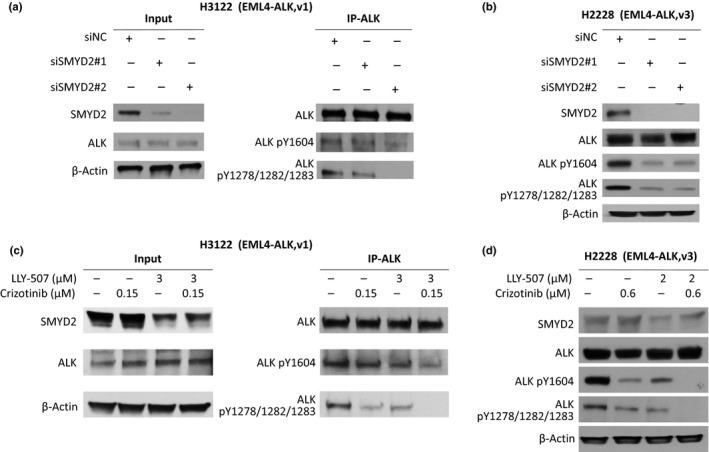
Knockdown of SMYD2 as well as treatment with a SMYD2 inhibitor or an anaplastic lymphoma kinase (ALK) inhibitor reduced phosphorylation levels of echinoderm microtubule‐associated protein‐like 4 (EML4)‐ALK fused proteins. (a, b) Western blots showing effects of SMYD2 knockdown on EML4‐ALK phosphorylation levels in NCI‐H3122 (a) and NCI‐H2228 (b) non‐small‐cell lung carcinoma cells. SMYD2 was knocked down with two different siRNAs (after 96 h of incubation). Proteins immunoprecipitated by anti‐ALK antibody or from whole cell lysis were subjected to gel electrophoresis. The samples were immunoblotted with anti‐SMYD2, anti‐ALK, anti‐phospho‐Tyr1604‐ALK (ALK pY1604), anti‐phospho‐ Tyr1278/1282/1283‐ALK (ALK pY1278/1282/1283), or anti‐β‐actin antibodies. (c, d) Western blots showing the pharmacologic effects of a SMYD2 inhibitor and/or an ALK inhibitor on EML4‐ALK phosphorylation in NCI‐H3122 (c) and NCI‐H2228 (d) cells. Cells were incubated with an ALK inhibitor (crizotinib) or/and a SMYD2 inhibitor (LLY‐507) for 6 h. Proteins immunoprecipitated by anti‐ALK antibody or from whole cell lysis were subjected to gel electrophoresis. The samples were immunoblotted with anti‐SMYD2, anti‐ALK, anti‐phospho‐Tyr1604‐ALK (ALK pY1604), anti‐phospho‐Tyr1278/1282/1283‐ALK (ALK pY1278/1282/1283), or anti‐β‐actin antibodies. IP, immunoprecipitant.

Phosphorylation of certain amino acid residues in ALK is known to be critically important in its oncogenic activity. Hence, we examined the effect of SMYD2‐mediated methylation of ALK on the phosphorylation status of EML4‐ALK fused protein using two NSCLC cell lines, NCI‐H2228 and NCI‐H3122, both of which express EML4‐ALK fused protein and also express high levels of SMYD2 protein endogenously. We knocked down SMYD2 expression in H2228 and H3122 cells using two siRNAs for SMYD2 (see sequence in Table S3), and examined EML4‐ALK phosphorylation status by Western blot analysis. As shown in Figure 2(a,b), knockdown of SMYD2 in H3122 and H2228 cells caused moderate (H3122) and drastic (H2228) decrease of phosphorylation levels at tyrosine 1278/1282/1283 as well as tyrosine 1604 of EML4‐ALK. To further validate the effect of SMYD2‐mediated methylation on the phosphorylation status of EML4‐ALK, we treated these two cell lines with a SMYD2‐specific inhibitor, LLY‐507, and compared the phosphorylation status of EML4‐ALK with the cells treated with an ALK inhibitor, crizotinib, or control. The results showed that the SMYD2 inhibitor LLY‐507 reduced phosphorylation levels of tyrosine 1278/1282/1283 and tyrosine 1604 of EML4‐ALK to levels similar to those produced by the ALK inhibitor crizotinib (Fig. 2c,d), implicating the significance of SMYD2‐mediated EML4‐ALK methylation on phosphorylation levels of tyrosine residues in EML4‐ALK that were known to be important for EML4‐ALK activity.

### Growth of NSCLC cells negatively affected by EML4‐ALK proteins with lysine 1610 substitution

**Figure 3:**
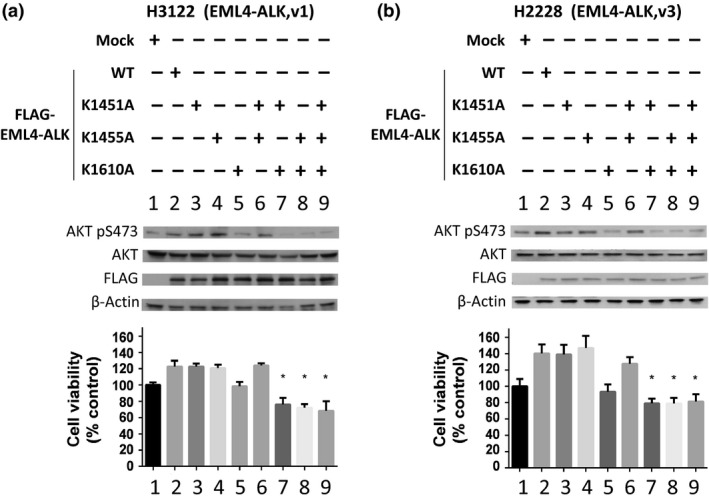
Methylation of anaplastic lymphoma kinase (ALK) at lysine 1610 dominantly promotes phosphorylation and activity of echinoderm microtubule‐associated protein‐like 4 (EML4)‐ALK in cancer cells. Upper panels, Western blots showing the effects of WT‐EML4‐ALK or K to A (lysine to alanine)‐substituted EML4‐ALK on phosphorylation of protein kinase B (AKT) in H3122 (a) and H2228 (b) non‐small‐cell lung carcinoma cells. H3122 and H2228 cells were separately transfected with Mock, WT‐, K1451A‐, K1455A‐, K1610A‐, K1451/1455A‐, K1451/1610A‐, K1455/1610A‐, or K1451/1455/1610A‐substituted EML4‐ALK incubated for 96 h. Proteins from whole cell lysis were subjected to gel electrophoresis. The samples were immunoblotted with anti‐SMYD2, anti‐AKT, anti‐phospho‐Ser473‐AKT antibody (AKT pS473) and anti‐β‐actin antibodies. Lower panels, growth‐promotive/suppressive effects of WT‐EML4‐ALK or K to A‐substituted EML4‐ALK proteins on H3122 (a) and H2228 (b) non‐small‐cell lung carcinoma. The relative cell numbers with the mean ± SD of three independent experiments were calculated. *P*‐values were calculated using Student's *t*‐test. *P < 0.05.

To further clarify the significance of SMYD2‐mediated methylation on EML4‐ALK protein on the growth of cancer cells, we examined the effect of exogenous introduction of lysine‐substituted EML4‐ALK proteins into two NSCLC cells with EML4‐ALK. We prepared eight plasmid clones designed to express a WT EML4‐ALK protein or seven lysine‐substituted EML4‐ALK proteins. Three clones contained a single substitution of lysine to alanine at codon 1451 (K1451A), 1455 (K1455A), or 1610 (K1610A), three clones contained double substitutions of K1451A/K1455A, K1451A/K1610A, or K1455A/K1610A, and one clone contained three lysine substitutions of K1451A/K1455A/K1610A. We transfected these eight plasmid clones separately into H3122 and H2228 cells, and measured the phosphorylation status of AKT, a downstream effector of activated EML4‐ALK protein. Transfection of WT EML4‐ALK or K1451A‐, K1455A‐, or K1451A/K1455A‐subsituted EML4‐ALK showed a slight increase of phosphorylation levels of AKT at serine 473 and some tendency of growth promotion for both cancer cells (not statistically significant). However, interestingly, transfection of four clones with lysine 1610 substitution on EML4‐ALK significantly reduced AKT phosphorylation levels (Fig. 3, upper panels). Concordant with the AKT phosphorylation levels, the viability of the two cell lines transfected with the clones with K1610A substitution was much lower than those transfected with other clones (Fig. 3, lower panels), indicating the dominant‐negative growth‐suppressive effect of proteins with K1610A substitution. Methylation at lysine 1610 of EML4‐ALK by SMYD2 is likely to be very important for the oncogenic activity of EML4‐ALK.

### Additive growth suppressive effect of dual ALK and SMYD2 inhibition on NSCLC cells with EML4‐ALK mutation

**Figure 4:**
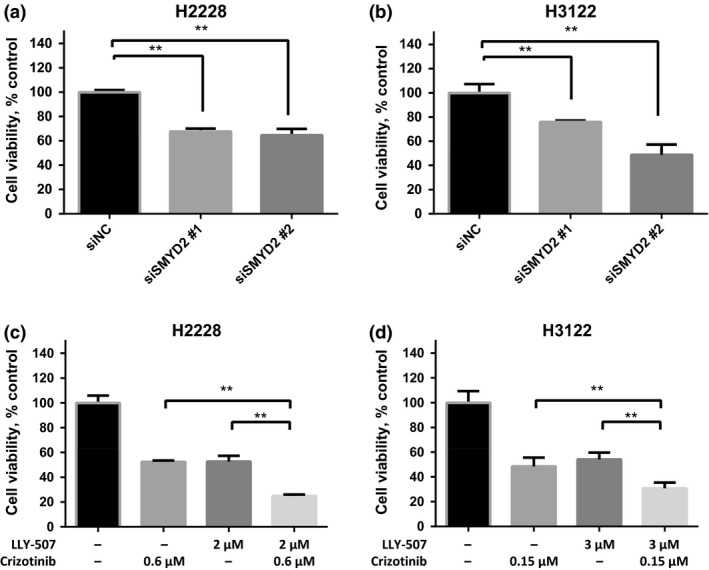
Growth suppressive effect of SMYD2 siRNAs and small‐molecule inhibitors for SMYD2 and/or anaplastic lymphoma kinase (ALK). (a, b) Significant growth suppressive effect of SMYD2 siRNA on H2228 (a) and H3122 (b) non‐small‐cell lung carcinoma cells. Relative cell numbers were calculated by a cell counting kit. (c, d) Significant growth suppressive effects of either or both crizotinib (ALK inhibitor) and LLY‐507 (SMYD2 inhibitor) on H2228 (c) and H3122 (d) cells. Cells were cultured with IC_50_ concentrations of either crizotinib (ALK inhibitor) and/or LLY‐507 (SMYD2 inhibitor) for 48 h, and the relative cell numbers were calculated using the cell counting kit. The mean ± SD of three independent experiments are shown. *P*‐values were calculated using Student's *t*‐test. **P < 0.01.

We then tested the effect of SMYD2 inhibition on the growth of H2228 and H3122 NSCLC cell lines. We knocked down SMYD2 expression using two siRNAs for SMYD2 (as shown in Fig. 2) and examined the viability of H2228 and H3122 cells, and found that knockdown of SMYD2 significantly suppressed the growth of both NSCLC cells (Fig. 4a,b). First, we found that IC_50_ values of LLY‐507 (SMYD2 inhibitor) for H2228 and H3122 cells were 2.0 and 3.0 μM, respectively, which were similar to four other NSCLC cell lines not expressing EML4‐ALK fused proteins (IC_50_ = 1.7–2.6 μM; Fig. S1). These findings indicated that SMYD2 might methylate multiple non‐histone proteins involved in proliferation/survival of NSCLC cells, as we reported previously, and treatment with SMYD2 inhibitor would be beneficial to NSCLC patients regardless of harboring EML4‐ALK fused proteins. To further investigate the additive effect of SMYD2 inhibition on an ALK inhibitor, we treated H2228 and H3122 cells with one or a combination of crizotinib (ALK inhibitor) and LLY‐507 at their IC_50_ concentration, and found that the combination of these two compounds additively suppressed the growth of the two cancer cell lines with EML4‐ALK fused protein (Fig. 4c,d).

## Discussion

Post‐translational modifications of various oncoproteins and tumor suppressors are known to play critical roles in tumorigenesis. Modulation of these modifications has been considered promising for the development of a novel class of anticancer treatment.^36^, ^37^, ^38^ Recent studies have shown that, in addition to epigenetic controls of gene expression through histone methylation, methylation of non‐histone protein also contributes to signaling transduction pathways, similar to other post‐translational modifications. We and others have reported the important roles of dysregulation of non‐histone protein methylation in carcinogenic processes in various types of human cancer. For example, SMYD3‐mediated AKT methylation at lysine 14 plays pivotal roles in activation of the AKT signaling pathway in breast and colon cancers,^16^ and PRMT1‐mediated methylation at arginine 887 of INCENP promotes mitosis of lung and cervical cancers.^39^

In the present study, we have shown that SMYD2‐mediated methylation of lysine 1610 on the EML4‐ALK fusion protein is likely to be critically important for autophosphorylation of some tyrosine residues and the oncogenic activity of the fused proteins. We identified three possible methylation candidate sites in ALK, K1451, K1455, and K1610, with an *in vitro* methyltransferase assay. To clarify the biological role of methylation at these residues, we constructed plasmid clones, each of which expressed EML4‐ALK protein with a substitution(s) of one, two, or all three lysine residues to an alanine. By exogenous introduction of these seven lysine to alanine‐substituted EML4‐ALK proteins into two NSCLC cells expressing endogenous EML4‐ALK protein, we found that four EML4‐ALK proteins, all of which included K1610A substitution commonly, revealed significant reduction of phosphorylation of serine 473 in AKT, one of the growth‐signaling molecules activated by EML4‐ALK, and showed the dominant‐negative growth‐suppressive effect on these two cancer cell lines. Hence, we assume that SMYD2‐mediated methylation of K1610, which is also physically close to the important phosphorylation site of tyrosine 1604 on ALK, may play a critical role in the oncogenic activity of EML4‐ALK. In addition, we showed that knockdown of SMYD2 abolished the *in vivo* phosphorylation of tyrosine 1278/1282/1283 as well as tyrosine 1604 in the two NSCLC cell lines that have an oncogenic *EML4‐ALK* fused gene.

Due to the intratumoral heterogeneity and the dynamic tumoral evolution during treatment, the clinical effect of monotherapies is generally limited and often not durable. Hence, combination chemotherapy is now applied as an effective strategy to improve the initial response of the treatment and to prevent relapse/recurrence caused by residual cancer cells that are probably resistant to a single agent.^40^ In the present study, although the biological mechanism for correlation between the K1610 methylation and Y1604 phosphorylation needs to be further clarified, our data imply the additive growth suppressive effect of the SMYD2 inhibitor to the ALK inhibitor through the enhanced downregulation of phosphorylation of EML4‐ALK fused protein and its downstream effector AKT. We also found that SMYD2 inhibitor (LLY‐507) showed similar growth‐suppressive effects in four NSCLC cell lines harboring *TP53* mutations but not harboring either EML4‐ALK fused proteins or genetic defects in the APC‐β‐catenin pathway. These findings suggested that SMYD2 might methylate multiple non‐histone proteins and activated several signal pathways involved in the proliferation/survival of NSCLC cells. Considering that several other oncoproteins in human tumors are also considered as SMYD2 substrates,^17^, ^41^, ^42^ targeting SMYD2 may be a promising approach to provide additive effects to other antitumor therapeutics.^10^

In conclusion, we identified EML4‐ALK as a novel substrate of the protein lysine methyltransferase SMYD2. This methylation might play a critical role in the phosphorylation of EML4‐ALK protein and its oncogenic activity. We also suggest that SMYD2 inhibitor might enhance the growth suppressive effect of ALK inhibitors.

## Disclosure Statement

Y. Nakamura is a stockholder and a scientific advisor of Oncotherapy Science, Inc. J. Park is a scientific advisor of Oncotherapy Science, Inc. The other authors have no conflict of interest.

**Abbreviations**
AKT: protein kinase B
ALK: anaplastic lymphoma kinase
EML4: echinoderm microtubule‐associated protein‐like 4
LC: liquid chromatography
MS: mass spectrometry
MS/MS: tandem mass spectrometry
MYPT1: myosin phosphatase target subunit 1
NSCLC: non‐small‐cell lung cancer
PRMT: protein arginine methyltransferase
SMYD3: SET and MYND domain‐containing protein 3
TKD: tyrosine kinase domain
WB: Western blot

## Supporting information


**Fig. S1**. Growth suppressive effect of LLY‐507 on non‐small‐cell lung carcinoma cell lines not harboring echinoderm microtubule‐associated protein‐like 4–anaplastic lymphoma kinase (EML4‐ALK) fusion protein.

**Table S1.** Information regarding non‐small‐cell lung carcinoma cell lines.

**Table S2.** Primer sequences used for construction of K‐A (lysine to alanine).

**Table S3.** Sequences of siRNA for SMYD2 and control siRNA.

## References

[1] Wang YC , Peterson SE , Loring JF . Protein post‐translational modifications and regulation of pluripotency in human stem cells. Cell Res 2014; 24: 143–60. 24217768 10.1038/cr.2013.151 PMC3915910

[2] Prabakaran S , Lippens G , Steen H , Gunawardena J . Post‐translational modification: Nature's escape from genetic imprisonment and the basis for dynamic information encoding. Wiley Interdiscip Rev Syst Biol Med 2012; 4: 565–83. 22899623 10.1002/wsbm.1185 PMC3473174

[3] Jenuwein T , Allis CD . Translating the histone code. Science 2001; 293: 1074–80. 11498575 10.1126/science.1063127

[4] Nakayama J , Rice JC , Strahl BD , Allis CD , Grewal SI . Role of histone H3 lysine 9 methylation in epigenetic control of heterochromatin assembly. Science 2001; 292: 110–3. 11283354 10.1126/science.1060118

[5] Garcia‐Cao M , O'Sullivan R , Peters AH , Jenuwein T , Blasco MA . Epigenetic regulation of telomere length in mammalian cells by the Suv39 h1 and Suv39 h2 histone methyltransferases. Nat Genet 2004; 36: 94–9. 14702045 10.1038/ng1278

[6] Bannister AJ , Kouzarides T . Regulation of chromatin by histone modifications. Cell Res 2011; 21: 381–95. 21321607 10.1038/cr.2011.22 PMC3193420

[7] Ruthenburg AJ , Allis CD , Wysocka J . Methylation of lysine 4 on histone H3: Intricacy of writing and reading a single epigenetic mark. Mol Cell 2007; 25: 15–30. 17218268 10.1016/j.molcel.2006.12.014

[8] Martin C , Zhang Y . The diverse functions of histone lysine methylation. Nat Rev Mol Cell Biol 2005; 6: 838–49. 16261189 10.1038/nrm1761

[9] Hamamoto R , Saloura V , Nakamura Y . Critical roles of non‐histone protein lysine methylation in human tumorigenesis. Nat Rev Cancer 2015; 15: 110–24. 25614009 10.1038/nrc3884

[10] Hamamoto R , Nakamura Y . Dysregulation of protein methyltransferases in human cancer: An emerging target class for anticancer therapy. Cancer Sci 2016; 107: 377–84. 26751963 10.1111/cas.12884 PMC4832871

[11] Cho HS , Shimazu T , Toyokawa G *et al* Enhanced HSP70 lysine methylation promotes proliferation of cancer cells through activation of Aurora kinase B. Nat Commun 2012; 3: 1072. 22990868 10.1038/ncomms2074 PMC3658001

[12] Cho HS , Suzuki T , Dohmae N *et al* Demethylation of RB regulator MYPT1 by histone demethylase LSD1 promotes cell cycle progression in cancer cells. Cancer Res 2011; 71: 655–60. 21115810 10.1158/0008-5472.CAN-10-2446

[13] Chuikov S , Kurash JK , Wilson JR *et al* Regulation of p53 activity through lysine methylation. Nature 2004; 432: 353–60. 15525938 10.1038/nature03117

[14] Huang J , Perez‐Burgos L , Placek BJ *et al* Repression of p53 activity by Smyd2‐mediated methylation. Nature 2006; 444: 629–32. 17108971 10.1038/nature05287

[15] Kunizaki M , Hamamoto R , Silva FP *et al* The lysine 831 of vascular endothelial growth factor receptor 1 is a novel target of methylation by SMYD3. Cancer Res 2007; 67: 10759–65. 18006819 10.1158/0008-5472.CAN-07-1132

[16] Yoshioka Y , Suzuki T , Matsuo Y *et al* SMYD3‐mediated lysine methylation in the PH domain is critical for activation of AKT1. Oncotarget 2016; 7: 75023–37. 27626683 10.18632/oncotarget.11898 PMC5342720

[17] Nakakido M , Deng Z , Suzuki T , Dohmae N , Nakamura Y , Hamamoto R . Dysregulation of AKT Pathway by SMYD2‐Mediated Lysine Methylation on PTEN. Neoplasia 2015; 17: 367–73. 25925379 10.1016/j.neo.2015.03.002 PMC4415136

[18] Soda M , Choi YL , Enomoto M *et al* Identification of the transforming *EML4‐ALK* fusion gene in non‐small‐cell lung cancer. Nature 2007; 448: 561–6. 17625570 10.1038/nature05945

[19] Takeuchi K , Choi YL , Soda M *et al* Multiplex reverse transcription‐PCR screening for EML4‐ALK fusion transcripts. Clin Cancer Res 2008; 14: 6618–24. 18927303 10.1158/1078-0432.CCR-08-1018

[20] Choi YL , Takeuchi K , Soda M *et al* Identification of novel isoforms of the *EML4‐ALK* transforming gene in non‐small cell lung cancer. Cancer Res 2008; 68: 4971–6. 18593892 10.1158/0008-5472.CAN-07-6158

[21] Doebele RC . A nice problem to have: When ALK inhibitor therapy works better than expected. J Thorac Oncol 2014; 9: 433–5. 24736061 10.1097/JTO.0000000000000124

[22] Katayama R , Shaw AT , Khan TM *et al* Mechanisms of acquired crizotinib resistance in ALK‐rearranged lung cancers. Sci Transl Med 2012; 4: 120ra17. 10.1126/scitranslmed.3003316 PMC3385512 22277784

[23] Kim S , Kim TM , Kim DW *et al* Heterogeneity of genetic changes associated with acquired crizotinib resistance in ALK‐rearranged lung cancer. J Thorac Oncol 2013; 8: 415–22. 23344087 10.1097/JTO.0b013e318283dcc0

[24] Choi YL , Soda M , Yamashita Y *et al* EML4‐ALK mutations in lung cancer that confer resistance to ALK inhibitors. N Engl J Med 2010; 363: 1734–9. 20979473 10.1056/NEJMoa1007478

[25] Mano H . Non‐solid oncogenes in solid tumors: EML4‐ALK fusion genes in lung cancer. Cancer Sci 2008; 99: 2349–55. 19032370 10.1111/j.1349-7006.2008.00972.x PMC11158085

[26] Li Y , Ye X , Liu J *et al* Evaluation of EML4‐ALK fusion proteins in non‐small cell lung cancer using small molecule inhibitors. Neoplasia 2011; 13: 1–11. 21245935 10.1593/neo.101120 PMC3022423

[27] Kwak EL , Bang YJ , Camidge DR *et al* Anaplastic lymphoma kinase inhibition in non‐small‐cell lung cancer. N Engl J Med 2010; 363: 1693–703. 20979469 10.1056/NEJMoa1006448 PMC3014291

[28] Chen J , Jiang C , Wang S . LDK378: A promising anaplastic lymphoma kinase (ALK) inhibitor. J Med Chem 2013; 56: 5673–4. 23837797 10.1021/jm401005u

[29] Shaw AT , Engelman JA . Ceritinib in ALK‐rearranged non‐small‐cell lung cancer. N Engl J Med 2014; 370: 2537–9. 10.1056/NEJMc1404894 24963575

[30] Shen L , Ji HF . Ceritinib in ALK‐rearranged non‐small‐cell lung cancer. N Engl J Med 2014; 370: 2537. 10.1056/NEJMc1404894 24963576

[31] Friboulet L , Li N , Katayama R *et al* The ALK inhibitor ceritinib overcomes crizotinib resistance in non‐small cell lung cancer. Cancer Discov 2014; 4: 662–73. 24675041 10.1158/2159-8290.CD-13-0846 PMC4068971

[32] Facchinetti F , Tiseo M , Di Maio M *et al* Tackling ALK in non‐small cell lung cancer: The role of novel inhibitors. Transl Lung Cancer Res 2016; 5: 301–21. 27413712 10.21037/tlcr.2016.06.10 PMC4931127

[33] Seto T , Kiura K , Nishio M *et al* CH5424802 (RO5424802) for patients with ALK‐rearranged advanced non‐small‐cell lung cancer (AF‐001JP study): A single‐arm, open‐label, phase 1‐2 study. Lancet Oncol 2013; 14: 590–8. 23639470 10.1016/S1470-2045(13)70142-6

[34] Vishwamitra D , Curry CV , Shi P , Alkan S , Amin HM . SUMOylation Confers Posttranslational Stability on NPM‐ALK Oncogenic Protein. Neoplasia 2015; 17: 742–54. 26476082 10.1016/j.neo.2015.09.005 PMC4611074

[35] Del Grosso F , De Mariano M , Passoni L , Luksch R , Tonini GP , Longo L . Inhibition of N‐linked glycosylation impairs ALK phosphorylation and disrupts pro‐survival signaling in neuroblastoma cell lines. BMC Cancer 2011; 11: 525. 22192458 10.1186/1471-2407-11-525 PMC3267831

[36] Tarhan YE , Kato T , Jang M *et al* Morphological Changes, Cadherin Switching, and Growth Suppression in Pancreatic Cancer by GALNT6 Knockdown. Neoplasia 2016; 18: 265–72. 27237318 10.1016/j.neo.2016.03.005 PMC4887616

[37] Vougiouklakis T , Sone K , Saloura V *et al* SUV420H1 enhances the phosphorylation and transcription of ERK1 in cancer cells. Oncotarget 2015; 6: 43162–71. 26586479 10.18632/oncotarget.6351 PMC4791223

[38] Vougiouklakis T , Hamamoto R , Nakamura Y , Saloura V . The NSD family of protein methyltransferases in human cancer. Epigenomics 2015; 7: 863–74. 25942451 10.2217/epi.15.32

[39] Deng X , Von Keudell G , Suzuki T *et al* PRMT1 promotes mitosis of cancer cells through arginine methylation of INCENP. Oncotarget 2015; 6: 35173–82. 26460953 10.18632/oncotarget.6050 PMC4742097

[40] Cunningham JJ , Gatenby RA , Brown JS . Evolutionary dynamics in cancer therapy. Mol Pharm 2011; 8: 2094–100. 21815657 10.1021/mp2002279 PMC3250072

[41] Hamamoto R , Toyokawa G , Nakakido M , Ueda K , Nakamura Y . SMYD2‐dependent HSP90 methylation promotes cancer cell proliferation by regulating the chaperone complex formation. Cancer Lett 2014; 351: 126–33. 24880080 10.1016/j.canlet.2014.05.014

[42] Cho HS , Hayami S , Toyokawa G *et al* RB1 methylation by SMYD2 enhances cell cycle progression through an increase of RB1 phosphorylation. Neoplasia 2012; 14: 476–86. 22787429 10.1593/neo.12656 PMC3394190

