# Supplementary material for: Effects of SMYD2‐mediated EML4‐ALK methylation on the signaling pathway and growth in non‐small‐cell lung cancer cells
Source: Cancer Sci. 2017 Jun 22;108(6):1203–9. doi: 10.1111/cas.13245 (PMC5480063; doi:10.1111/cas.13245)
Supplement: Supplementary file 1 — Fig. S1. Growth suppressive effect of LLY‐507 on non‐small‐cell lung carcinoma cell lines not harboring echinoderm microtubule‐associated protein‐like 4–anaplastic lymphoma kinase (EML4‐ALK) fusion protein. [file CAS-108-1203-s001.docx]

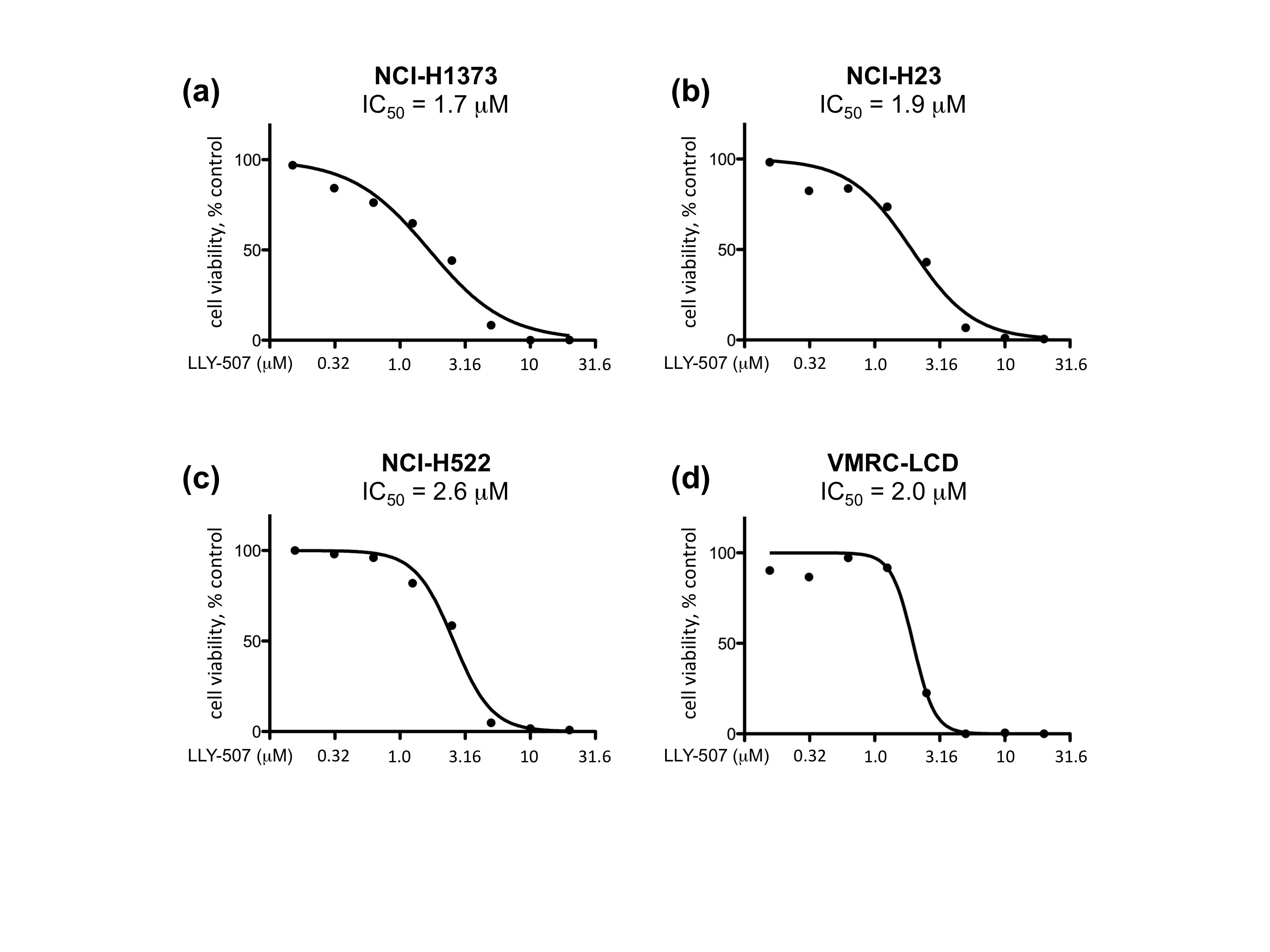


Fig. S1. Growth suppressive effect of LLY-507 on NSCLC cell lines not harboring EML4-ALK fusion protein. Graphs indicate anti-proliferative effects and IC_50_ values of LLY-507 in 4 NSCLC cell lines, NCI-H1373 (**a**), NCI-H23 (**b**), NCI-H522 (**c**), and VMRC-LCD (**d**). The cells were cultured with LLY-507 at indicated concentrations for 72 hrs, and the relative cell numbers were calculated using the CCK8 kit.
