# Supplementary material for: Effects of SMYD2‐mediated EML4‐ALK methylation on the signaling pathway and growth in non‐small‐cell lung cancer cells
Source: Cancer Sci. 2017 Jun 22;108(6):1203–9. doi: 10.1111/cas.13245 (PMC5480063; doi:10.1111/cas.13245)
Supplement: Supplementary file 2 — Table S1. Information regarding non‐small‐cell lung carcinoma cell lines. [file CAS-108-1203-s002.docx]

Table S1: Information of cell lines.

| Name | Origin | Certification from | Tested methods | DNA profile or characteristics |
| --- | --- | --- | --- | --- |
| 293T | Human embryonic kidney fibroblast | ATCC | STR | Amelogenin:X CSF1PO:11,12 D13S317:12,14 D16S539:9,13 D5S818:8,9 D7S820:11 TH01:7,9.3 TPOX:11 vWA:16,18,19 |
| NCI-H2228 | Human non-small cell lung adenocarcinoma | ATCC | STR | Amelogenin:X CSF1PO: 12 D13S317: 11 D16S539: 11,13 D5S818: 12 D7S820:11 TH01: 7,8 TPOX:11  vWA: 15,17 |
| NCI-H3122 | Human non-small cell lung adenocarcinoma | ATCC | STR | Amelogenin:X CSF1PO: 11,12 D13S317: 10,12 D16S539: 11,12 D5S818: 11,12 D7S820:8,12 TH01:7,9.3 TPOX: 10  vWA: 16 |
