# Supplementary material for: Effects of SMYD2‐mediated EML4‐ALK methylation on the signaling pathway and growth in non‐small‐cell lung cancer cells
Source: Cancer Sci. 2017 Jun 22;108(6):1203–9. doi: 10.1111/cas.13245 (PMC5480063; doi:10.1111/cas.13245)
Supplement: Supplementary file 3 — Table S2. Primer sequences used for construction of K‐A (lysine to alanine). [file CAS-108-1203-s003.docx]

Table S2: Primer sequences used for construction of K-A (lysine to alanine) substituted expression vectors

| Substitution | Primer sequences | |
| --- | --- | --- |
| ALK-TKD-K1451A | | F 5'- CTGGCGCGGCTGCAAAGAAACCCAC-3' |
|  |  | R 5'- AGGAGGTGGTAGGCAGAGGTGGTGG-3' |
| ALK-TKD-K1455A | | F 5'- CTGGCAAGGCTGCAAAGGCACCCAC-3' |
|  |  | R 5'- AGGAGGTGGTAGGCAGAGGTGGTGG-3' |
| ALK-TKD-K1610A | | F 5'- ACCATTCTGGCAAGCAAGAATAGC-3' |
|  |  | R 5'- ATCCTCGTAATGACCAGCTCCAGG-3' |
| EML4-ALK-K1451A | | F 5'- CTACCACCTCCTCTGGCGCGGCTGCAAAGAAAC -3' |
|  |  | R 5'-  GCAGAGGTGGTGGGGCAGCTGGGCTGCGCTCCTCCTC-3' |
| EML4-ALK-K1455A | | F 5'-CAAAGGCACCCACAGCTGCAGAGGTCTCTGTTCGAGT-3' |
|  |  | R 5'- CAGCCTTGCCAGAGGAGGTGGTAGG-3' |
| EML4-ALK-K1610A | | F 5'- TTCTGGCAAGCAAGAATAGCATGAAC-3' |
|  |  | R 5'- TGGTATCCTCGTAATGACCAGCTCCAGGGGCAGTAG-3' |
| EML4-ALK-K1451/1455A | | F 5'- CTCTGGCGCGGCTGCAAAGGCACCCACAGCTGCAG -3' |
|  |  | R 5'- GAGGTGGTAGGCAGAGGTGGTGGGG -3' |
