# Supplementary material for: Effects of SMYD2‐mediated EML4‐ALK methylation on the signaling pathway and growth in non‐small‐cell lung cancer cells
Source: Cancer Sci. 2017 Jun 22;108(6):1203–9. doi: 10.1111/cas.13245 (PMC5480063; doi:10.1111/cas.13245)
Supplement: Supplementary file 4 — Table S3. Sequences of siRNA for SMYD2 and control siRNA. [file CAS-108-1203-s004.docx]

Table S3: Sequences of siRNA for SMYD2 and control siRNA

| **siRNA name** | **Sequences** |
| --- | --- |
| siNC (cocktail) | 5’-AUCCGCGCGAUAGUACGUA-3’ |
|  | 5’-UUACGCGUAGCGUAAUACG-3’ |
|  | 5’-UAUUCGCGCGUAUAGCGGU-3’ |
| siSMYD2#1 | 5’-GAUUUGAUUCAGAGUGACA-3’ |
| siSMYD2#2 | 5’-GAAAUGACCGGUUAAGAGA-3’ |
